# Configuring reconfigurable intelligent surfaces using a practical codebook approach

**DOI:** 10.1038/s41598-023-31596-7

**Published:** 2023-07-22

**Authors:** Saber Hassouna, Muhammad Ali Jamshed, Masood Ur-Rehman, Muhammad Ali Imran, Qammer H. Abbasi

**Affiliations:** James Watt School of Engineering, University of Glasgow, Glasgow, UK

**Keywords:** Energy science and technology, Engineering

## Abstract

It is proven that the scattering, reflection, and refraction properties of electromagnetic signals can be adapted and managed by using reconfigurable intelligent surfaces (RISs). In this paper, we have investigated the performance of a single-input-single-output (SISO) wideband system in terms of achievable data rate by optimizing the phases of RIS elements and performing a fair power allocation for each subcarrier over the entire bandwidth. A new beamforming codebook is developed from which the maximizing signal-to-noise (SNR) configuration is selected. The channel state information (CSI) along with the selected maximizing SNR configuration is then used by the proposed power algorithm to obtain the optimal configuration of the RIS. To validate our proposed method, it is compared with state-of-the-art semidefinite relaxation (SDR) scheme in terms of performance, complexity and run-time consumption. Our method shows dramatically lower computational complexity than the SDR method and achieves an order of 2.5 increase in the achievable data rate with an optimized RIS compared with an un-configured surface.

## Introduction

Reconfigurable intelligent surfaces (RISs) have recently emerged as a novel technique to improve wireless channel characteristics^1–3^. Signal concentration at the receiver is possible because of the constructive superposition of the line-of-sight (LoS) route between the transmitter and the receiver and the reflections from all RIS elements, which together create a beam in a specific direction^4^. RISs differ from wireless repeaters and relays in several ways, including the fact that they may be configured, and do not need signal amplification, complex processing, coding, and decoding methods^5^.

Common methods employed in the current research contributions for optimizing the passive beamforming, such as semidefinite relaxation (SDR)^6^, strongest signal path maximization in time domain^2,7^ and successive convex approximations^8^. Applying the SDR and the successive convex approximation approaches require efficient convex optimization tools. Furthermore, the SDR needs Gaussian randomization methods to obtain the rank-one suboptimal solution. Such great efforts adopted complex algorithms to find high-quality solutions near the optimal. However, the computational complexity is high and is seen as practically expensive when the number of RIS elements is fairly large, which corresponds to the situation when the RIS is most beneficial.

In this paper, we summarize our main contributions as follows:

- We have maximized the achievable data rate by optimizing the allocated power for every subcarrier and the phases of the RIS elements. The passive beamforming design is still a non-trivial task to tackle. We propose an iterative power method to optimize the reflection coefficient phases of the RIS elements. Then, we allocate the power for each orthogonal frequency division multiplexing (OFDM) subcarrier taking into consideration its channel gain status by using the famous water-filling algorithm.
- It is noted that for large surfaces the reflection coefficients can be estimated by the column of the discrete Fourier transform (DFT) matrix^9^. Similarly, we have used pre-designed phase shift configurations generated by our extended-designed beamforming codebook approach to configuring the RIS. We initialize the power method with maximizing signal-to noise ratio (SNR) configuration selected from the codebook to compute the dominant eigenvector of the channel power and hence, increase the achievable data rate.
- The performance of the power method is validated by comparing it with the SDR method. The achievable data rate is studied in the low and high SNR regimes for both methods under different RIS sizes (varying the number of RIS elements).
- Computational complexity and run-time analysis of the proposed power method are compared against the SDR method. Simulation results show that the proposed power method achieves a very close achievable rate in comparison to the benchmark scheme, but with significantly lower computational complexity.

The rest of the paper is organized as follows. Section “System model” presents the system model. In “RIS configuration for data rate maximization” Section the RIS configuration for data rate maximization is studied. The simulation results are discussed in “Simulation results” Section while the conclusion is given in “Conclusion” Section.

## System model

**Figure 1:**
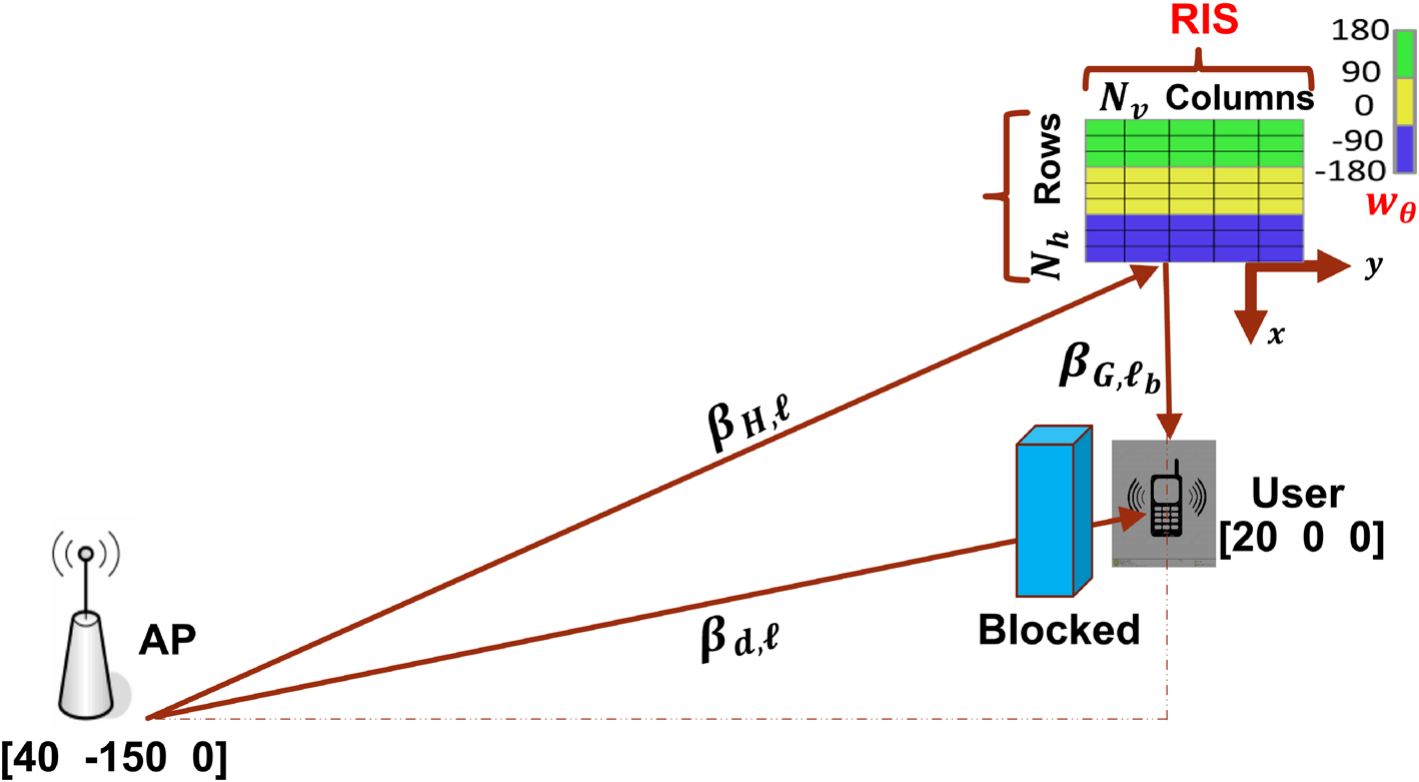
System setup illustration.

A single-input-single-output (SISO) wideband model where a single antenna (transmitter) communicates with a single user (receiver) is considered. A RIS with *N* reconfigurable elements is considered within the coverage region of the transmitter and the receiver as shown in Fig. 1. The transmission is carried out using OFDM. Let *s*(*k*) refer to the transmitted discrete-time signal in the complex baseband domain so, the discrete-time signal received at the receiver can be represented as^2^:

1 $$\begin{aligned} y[k]=\sum _{l=0}^{M-1} h_\theta [l] s[k-l]+e[k], \end{aligned}$$

where $\left\{ h_\theta [l]: l=0, \ldots , M-1\right\}$ is the wideband channel in the time domain with the RIS configuration $\theta$ and $e[k] \sim \mathcal {N}_{\mathbb {C}}\left( 0, \sigma ^2\right)$ is the receiver noise. $h_d[l]$ is given by:

2 $$\begin{aligned} h_\theta [l]=h_d[l]+u_l^T w_\theta , \end{aligned}$$

where $h_d[l]$ is the access point (AP)–User uncontrollable direct channel, $u_l \in \mathbb {C}^N$ is the AP-RIS-User controllable indirect channels via all RIS *N* elements and the reflection coefficients of all elements is denoted by $w_\theta \in \mathbb {C}^N$. OFDM transmission with $K>M$ subcarriers and cyclic prefix length $M-1$ is considered. Consequently, time domain signals with a block of $K+M-1$ are sent to generate a block of OFDM signal that contains *K* of parallel subcarriers by applying the DFT:

3 $$\begin{aligned} \bar{y}[\nu ]=\bar{h}_\theta [\nu ] \bar{s}[\nu ]+\bar{e}[\nu ], \quad \nu =0, \ldots , K-1. \end{aligned}$$

The system model in (3) can be illustrated in vector form as follows:

4 $$\begin{aligned} \left[ \begin{array}{c} \bar{y}[0] \\ \vdots \\ \bar{y}[K-1] \end{array}\right] =\left[ \begin{array}{c} \bar{h}_\theta [0] \\ \vdots \\ \bar{h}_\theta [K-1] \end{array}\right] \odot \left[ \begin{array}{c} \bar{s}[0] \\ \vdots \\ \bar{s}[K-1] \end{array}\right] +\left[ \begin{array}{c} \bar{e}[0] \\ \vdots \\ \bar{e}[K-1] \end{array}\right] , \end{aligned}$$

where $\odot$ describes the Hadamard product. The channel vector $\bar{h}_\theta$ can be realized a function of $w_\theta$ as per following:

5 $$\begin{aligned} \bar{h}_{\theta }=F\left[ \begin{array}{c} h_{d}[0]+u_{0}^{T} w_{\theta } \\ \vdots \\ h_{d}[M-1]+u_{M-1}^{T} w_{\theta } \end{array}\right] =F\left( h_{d}+U^{T} w_{\theta }\right) , \end{aligned}$$

where, $h_{d}=\left[ h_{d}[0], \ldots , h_{d}[M-1]\right] ^{T}$ represents the AP-User uncontrollable direct channel components, $U=\left[ u_{0}, \ldots , u_{M-1}\right] \in \mathbb {C}^{N \times M}$ contains all the components of the AP-RIS-User controllable indirect propagation channels. The perfect channel model is assumed to be available in the system. *F* is a $K \times M$ DFT matrix with the (*v*, *k*)*th* element being $e^{-\frac{2 \pi k v}{K}}$ and $w_\theta =\left[ w_{\theta _1}, \ldots , w_{\theta _N}\right] ^T \in \mathbb {C}^{N \times 1}$ contains the reflection coefficients of the RIS that accounts for the effective phase shifts $\left( \theta _1, \theta _2, \ldots , \theta _N\right) \in [-\pi , \pi )$ and amplitude coefficients $\left( \gamma _1, \gamma _2, \ldots , \gamma _N\right) \in [0,1]$. Each RIS element in a one-bit RIS design can switch between two states of phase shifts, $\pi /2$ or $-\pi /2$. We inspired the generation of such phase matrices from the beamforming codebook of the RIS presented in^9^. Consequently, we adopted a practical discrete phase model for all RIS elements rather than continuously adjusted phase shifts. The sum rate over the *K* subcarriers, for a given RIS configuration, assuming equal power allocation and under perfect channel at the receiver is given as:

6 $$\begin{aligned} R=\frac{B}{K+M-1} \sum _{v=0}^{K-1} \log _{2}\left( 1+\frac{P_{v}\left| f_{v}^{H} h_{d}+f_{v}^{H} U^{T} w_{\theta }\right| ^{2}}{B N_{o}}\right) \frac{b i t}{s}, \end{aligned}$$

where *B* is the channel bandwidth, $P_{v}$ is the transmit power assigned for subcarrier *v*, *M* is the number of channel taps, $f_{v}^{H}$ is the *v* *th* row of the DFT matrix *F*. The upper bound for (6) is given by^2^:

7 $$\begin{aligned} R \le \frac{B}{K+M-1} \sum _{v=0}^{K-1} \log _{2}\left( 1+\frac{P_{v}}{B N_{o}}\left( \left| f_{v}^{H} h_{d}\right| +\left\| f_{v}^{H} U^{T}\right\| _{1}\right) ^{2}\right) , \end{aligned}$$

where $\Vert \cdot \Vert _{1}$ is the $L_{1}$ norm.

### Wideband channel model

The following wideband channel is considered^10^:

8 $$\begin{aligned} h_d=\sum _{\ell =1}^{L_d} \sqrt{\beta _{d, \ell }} e^{-j 2 \pi f_c \tau _{d, \ell }}\left[ \begin{array}{c} {\mathrm {sinc}}\left( 0+B\left( \alpha -\tau _{d, \ell }\right) \right) \\ \vdots \\ {\mathrm {sinc}}\left( M-1+B\left( \alpha -\tau _{d, \ell }\right) \right) \end{array}\right] , \end{aligned}$$

where, $L_d$ is the number of propagation paths, $B_{d, \ell } \ge 0$ is the pathloss of the $\ell$th path from the AP to the user as per Fig. 1, $\tau _{d, \ell }$ is the propagation delay and $\alpha$ is the sampling delay over the shortest path. Similarly, the controllable path is given by:

9 $$U = \sum\limits_{{\ell = 1}}^{{L_{a} }} {\sum\limits_{{\ell _{b} = 1}}^{{L_{b} }} {\sqrt {\beta _{{H,\ell }} \beta _{{G,\ell _{b} }} } } } e^{{ - j2\pi f_{c} \left( {\tau _{{H,\ell }} + \tau _{{G,\ell _{b} }} } \right)}} \left( {a\left( {\varphi _{{H,\ell }} ,\theta _{{H,\ell }} } \right) \odot a\left( {\varphi _{{G,\ell }} ,\theta _{{G,\ell _{b} }} } \right)} \right)\left[ {\begin{array}{*{20}c} {{\mathrm{sinc}}\left( {0 + B\left( {\alpha - \tau _{{H,\ell }} - \tau _{{G,\ell }} } \right)} \right)} \\ \vdots \\ {{\mathrm{sinc}}\left( {M - 1 + B\left( {\alpha - \tau _{{H,\ell }} - \tau _{{G,\ell _{b} }} } \right)} \right)} \\ \end{array} } \right]^{T}$$

The propagation paths $L_a$ and $L_b$ are from the AP to the RIS and from the RIS to the users, respectively. $B_{H, \ell } \ge 0$, and $B_{G, \ell _b} \ge 0$, are the pathlosses from the AP to the RIS and from the RIS to the user, as per Fig. 1. $\tau _{H, \ell }$ and $\tau _{G, \ell _b}$ are the propagation delays to and from the RIS and $a(\varphi , \theta )=\sqrt{\mathcal {G}(\varphi , \theta )}\left[ e^{j \mathcal {K}(\varphi , \theta )^T} \mathbb {U}_1, \ldots , e^{j \mathcal {K}(\varphi , \theta )^T} \mathbb {U}_N\right] ^T$ is the array response vector. Where $\mathcal {G}(\varphi , \theta )$ is the directivity pattern for each element, $\theta$ and $\varphi$ are the elevation and azimuth angles, $\mathcal {K}(\varphi , \theta )=\frac{2 \pi }{\lambda }[\cos (\theta ) \cos (\varphi ), \cos (\theta ) \sin (\varphi ), \sin (\theta )]^T$ is the wave vector, $\mathbb {U}_m=[0, i(m) 0.25 \lambda , j(m) 0.25 \lambda ]^T$ is the location of the $m^{\mathrm {th}}$ element with horizontal $i(m)=\bmod \left( m-1, N_h\right)$ and vertical $j(m)=\left\lfloor (m-1) / N_h\right\rfloor$ indices of element *m*, mod(., .) and $\lfloor .\rfloor$ are referring to the modulus and truncate operations respectively. The vertical and the horizontal spacing of elements are $\lambda / 4$ with $\lambda =0.1$ m. The direct $h_{d}$ and indirect channels *U* are assumed to be known at the receiver.

## RIS configuration for data rate maximization

The wideband case with *K* orthogonal subcarriers presents a more difficult RIS optimization problem than narrowband (containing a single subcarrier). The maximization of (6) requires estimating the optimal vector $w_{\theta }$ and fairly allocating power for each subcarrier.

### Beamforming codebook

**Figure 2:**
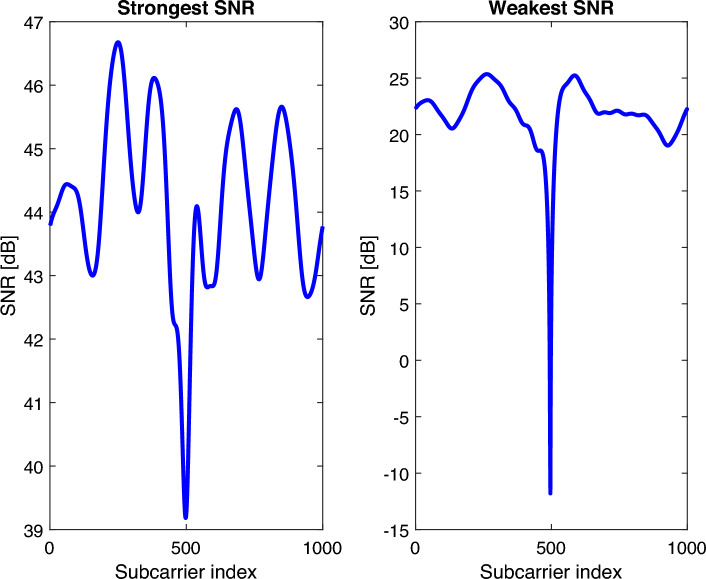
The best configuration in the codebook that gives the maximum SNR and the worst configuration that gives the minimum SNR.


For large value of *N*, there are $2^{N}$ possible RIS configurations and requires extensive analysis to find the optimal configuration, which is practically unattainable. Existing research relies on accurate configurations, in which a RIS with *N* reflecting elements may swap between *N* orthogonal configurations. For a particular incident and desirable reflected angles, the structure of the optimal RIS phase shifts remains consistent with the 2D-DFT codebook^9^. Consequently, each column of the codebook beamformer $W_{\theta }=F\left( N_{v}\right) \otimes F\left( N_{h}\right) \in \mathbb {C}^{N \times N}$, $\otimes$ refer to the kronecker product, can be a possible reflection configuration for an incident signal in a certain beam direction. We have extended $W_{\theta }$ to $W_{\theta _{4 N}}$ where $W_{\theta _{4 N}}=\left[ W_{\theta },-W_{\theta }, W_{(\theta , \mathrm {flip})},-W_{(\theta , \mathrm {flip})}\right] \in \mathbb {C}^{N \times 4 N}$, to invert the phases of columns and rows in a way to increase the varieties of the codebook phase configurations. $W_{(\theta , f l i p)}$ denotes the output matrix when every column is flipped upside-down. For such a codebook setting, the probability of getting a strong SNR configuration is very high. The DFT matrices for the columns $F\left( N_{v}\right)$ and $F\left( N_{h}\right)$ can be denoted as:

10 $$\begin{aligned} F\left( N_{v}\right) =\left[ \begin{array}{ccccc} 1 &{} 1 &{} 1 &{} \cdots &{} 1 \\ 1 &{} f_{N_{v}} &{} f_{N_{v}}^{2} &{} \cdots &{} f_{N_{v}}^{N_{v}-1} \\ \vdots &{} \vdots &{} \vdots &{} \vdots &{} \vdots \\ 1 &{} f_{N_{v}}^{N_{v}-1} &{} f_{N_{v}}^{2\left( N_{v}-1\right) } &{} \cdots &{} f_{N_{v}}^{\left( N_{v}-1\right) \left( N_{v}-1\right) } \end{array}\right] , \end{aligned}$$

where $f_{N_{v}}=e^{-j 2 \pi / N_{v}}=\cos \left( 2 \pi / N_{v}\right) -\sin \left( 2 \pi / N_{v}\right)$). $N_{v}$ and $N_{h}$ are the vertical and horizontal elements of RIS, as illustrated in Fig. 1. The phase shifts generated by the codebook must be quantized to meet the design requirements of the RIS. As a result, the reflection coefficient $w_{\theta }$ for element *i* can be either $e^{j \pi / 2}$ if $\arg \left( \left[ w_{\theta }\right] _{i}\right) \in [-\pi , 0)$ or $e^{-j \pi / 2}$ if $\arg \left( \left[ w_{\theta }\right] _{i}\right) \in [0, \pi )$^9^. Let us denote the best phase configuration $w_{\theta _{\mathrm{ CodeBook } }} \in W_{\theta _{4 N}}$ that can be generated from the codebook. It is considered as the benchmark for obtaining the maximum SNR.

11 $$\begin{aligned} S N R=\frac{P\left| \bar{h}_{\theta }\right| ^{2}}{B N_{o}}, \end{aligned}$$

where, *P* is the power for all subcarriers. We search in the codebook $W_{\theta _{4 N}}$ for the best configuration that maximize the SNR in (11). Figure 2 depicts the related strongest and weakest SNR with a difference of around 20 dB. The strongest SNR configuration is further utilized in Algorithm 1.

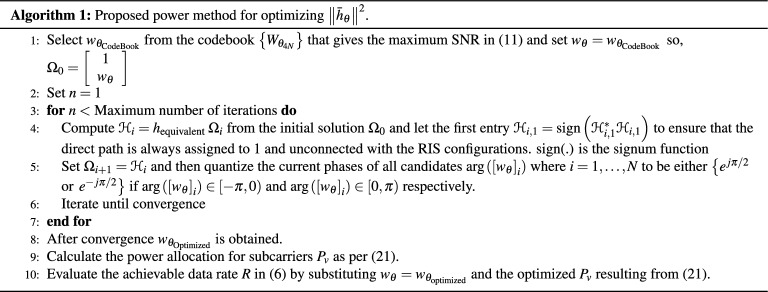


### Problem formulation

In this paper, we optimize the transmit power *P* and the reflection coefficients vector $w_{\theta }$ for all RIS elements to maximize the sum rate at the receiver. The sum rate maximization problem can be mathematically represented in terms of optimization problem (OP) as follows:

12 $$\begin{gathered} {\mathrm{(OP)}1}\mathop {{\mathrm{max}}}\limits_{{{P},{w_{\theta } }}} \quad R({P}, {w_{\theta }} ) = \sum\limits_{{v = 0}}^{{K - 1}} {\log _{2} } \left( {1 + \frac{{P_{v} \left| {f_{v}^{H} h_{d} + f_{v}^{H} U^{T} w_{\theta } } \right|^{2} }}{{BN_{o} }}} \right), \hfill \\ {\mathrm{s}}.{\mathrm{t}}: \hfill \\ \;(C1):|[w_{\theta } ]_{i} | = 1,\quad \forall i \in N, \hfill \\ \;(C2):\frac{1}{K}\sum\limits_{{v = 0}}^{{K - 1}} {P_{v} } \le P, \hfill \\ \;(C3):P_{v} \ge 0\quad \quad\;\; \forall _{v} \in K. \hfill \\ \end{gathered}$$

Constraint (C1) ensures that each RIS element’s reflection has no pathloss. Unlike relays, RIS elements do not amplify or decode, necessitating the use of unit magnitude elements. Constraint (C2) confirms that the power allocated for all subcarriers should not exceed the base station power budget and must be $\ge$ 0, as per Constraint (C3). It is noted that the (OP1) is non-convex over the unit modular (C1) on all RIS elements^8^. We overcome the non-convexity by iteratively applying the power method until it converges to the most preferred $w_{\theta }$ that will affect all number of subcarriers. Moreover, the power is allocated to each subcarrier in (C2) by using the well-known water-filling algorithm.

### Proposed solution

#### Power method

We use the power method to find a low-complex solution to optimize the RIS phase shifts. It calculates the dominant eigen vector of the channel power. The (OP1) problem in (12) can be simplified to be equivalent to the following channel gain:

13 $$\begin{gathered} ({\mathrm{OP}}2)\max _{{w_{\theta } }} R\left( {w_{\theta } } \right) = \sum\limits_{{v = 0}}^{{K - 1}} {\left( {\left| {f_{v}^{H} h_{d} + f_{v}^{H} V^{T} w_{\theta } } \right|^{2} } \right)} \hfill \\ {\mathrm{s}}.{\mathrm{t}}: \hfill \\ \;(C1):\left| {\left[ {w_{\theta } } \right]_{i} } \right| = 1,\forall i \in N. \hfill \\ \end{gathered}$$

Thus the summation of the objective function of (13) for the entire number of subcarriers $(v=0,1, \ldots , K-1)$ is denoted by the channel gain $\left| \bar{h}_\theta \right| ^2$:

14 $$\begin{aligned} \left\| \bar{h}_\theta \right\| ^2=\underbrace{\left[ \begin{array}{c} 1 \\ w_\theta \end{array}\right] ^H}_{\Omega ^H} \underbrace{\left[ h_d, U^T\right] ^H\left[ h_d, U^T\right] }_{h_{\mathrm{ equivalent } }} \underbrace{\left[ \begin{array}{c} 1 \\ w_\theta \end{array}\right] }_{\Omega }. \end{aligned}$$

We select the $w_{\theta _{\mathrm {CodeBook}}}$ from the codebook $W_{\theta _{4 N}} \in \mathbb {C}^{N \times 4 N}$ to initialize the power method to maximize the quadratic form of the channel $\left\| \bar{h}_{\theta }\right\| ^{2}$ as shown in Algorithm 1. The power method finds the dominant eigenvalue by iterating the computation of $\Omega _{i+1}=\frac{h_{\mathrm {equivalent}} \Omega _{i}}{\left\| h_{\mathrm {equivalent}} \Omega _{i}\right\| }$ from the initial solution $$\Omega _0=\left[ \begin{array}{c}1 \\ w_\theta \end{array}\right]$$ where we set $w_\theta =w_{\theta _{\mathrm {CodeBook}}}$ until convergence. In each step we find a vector of $w_\theta$ solution and then, we project it to the closest one-bit RIS phases by quantizing the vector entries of phases to be either $\pi / 2$ or $-\pi / 2$.

#### Semidefinite relaxation method

The SDR is a widely used technique for dealing with the non-convex unit-modulus constraint by converting the passive beamforming vector $w_\theta$ into a rank-one and positive semi-definite matrix. The initial non-convex problem is converted into a convex Semidefinite Program (SDP) problem using the SDR method, which may then be addressed by a variety of effective convex optimization tools. The (OP2) objective function of (13) is described as:

15 $$\begin{aligned} \widetilde{\mathfrak {U}}=\sum _{v=0}^{K-1} f_v^H U U^H, \end{aligned}$$

16 $$\begin{aligned} \tilde{u}=\sum _{v=0}^{K-1} f_v^H U h_d, \end{aligned}$$

17 $$\begin{aligned}\tilde{ \mathfrak{D}}=\sum _{v=0}^{K-1}\left| f_v^H h_d\right| ^2, \end{aligned}$$

so, (OP2) is equivalent to:

18 $$\begin{gathered} ({\mathrm{OP}}3):\max _{{w_{\theta } }} \left( {w_{\theta }^{H} \widetilde{{\mathfrak{U}}}w_{\theta } + w_{\theta }^{H} \tilde{u} + u^{H} w_{\theta } + \widetilde{{\mathfrak{D}}}} \right), \hfill \\ {\mathrm{s}}.{\mathrm{t}}: \hfill \\ \left| {\left[ {w_{\theta } } \right]_{i} } \right| = 1,\forall i = 1,2, \ldots ,K. \hfill \\ \end{gathered}$$

It is simple to identify problem (OP3) as a quadratically constrained quadratic program (QCQP) problem that can be expressed as a homogeneous QCQP problem, particularly, by denoting:

19 $$\begin{aligned} \mathfrak {Q}=\left[ \begin{array}{cc} \widetilde{\mathfrak {U}} &{} \tilde{u} \\ \widetilde{u}^H &{} 0 \end{array}\right] , \quad \widetilde{w}_\theta =\left[ \begin{array}{c} w_\theta \\ t \end{array}\right] , \end{aligned}$$

where, *t* is an auxiliary variable and $\phi =\widetilde{w}_\theta \widetilde{w}_\theta ^T$. Optimization Problem (OP3) is transformed into the following problem:

20 $$\begin{gathered} ({\mathrm{OP}}4):\max _{\phi } {\mathrm{tr}}({\mathfrak{Q}}\phi ) + \widetilde{{\mathfrak{D}}}, \hfill \\ {\mathrm{s}}.{\mathrm{t}}: \hfill \\ [\phi ]_{{i,i}} = 1,\forall i = 1,2, \ldots ,N + 1, \hfill \\ \phi \ge 0. \hfill \\ \end{gathered}$$

It should be noticed that (OP4) is still non-convex because of the rank-one constraint. The rank-one constraint is thus bypassed by using the SDR approach, which converts (OP4) into a convex SDP that can be successfully solved using modern convex optimization solvers like CVX^11^. It is essential to keep in mind that the optimum objective value of (OP4) serves as an upper bound on that of (OP2). However, the best solution $\phi ^*$ to (OP4) might not be a rank-one answer. As a result, we arrive at the following rank-one solution using the Gaussian randomization method $\phi ^*=\Lambda \Sigma \Lambda ^H$. Where, $\Lambda =\left[ \rho _1, \cdots , \rho _{N+1}\right]$ and $\Sigma ={\mathrm {diag}}\left( \sigma _1, \cdots , \sigma _{N+1}\right)$ are unitary and diagonal matrices, respectively, both with the size of $(N+1) \times (N+1)$. We get sub-optimal solution to (OP4) , $\widetilde{w}_\theta ^*=\Lambda \Sigma r$, where $r \in \mathbb {C}^{(N+1) \times 1}$ is a random vector which is generated based on $r \in \mathcal {N}_{\mathbb {C}}\left( 0, I_{N+1}\right)$ with zero mean circularly symmetric complex Gaussian (CSCG) distribution and covariance matrix $I_{N+1}$. We eventually arrive at suboptimal solution to (OP2) as $w_\theta ^*=e^{j \angle \left( \left[ \tilde{w}_\theta ^*\right] _{1: N} /\left[ \tilde{w}_\theta \right] _{N+1}\right) }$.

#### Water-filling algorithm

**Figure 3:**
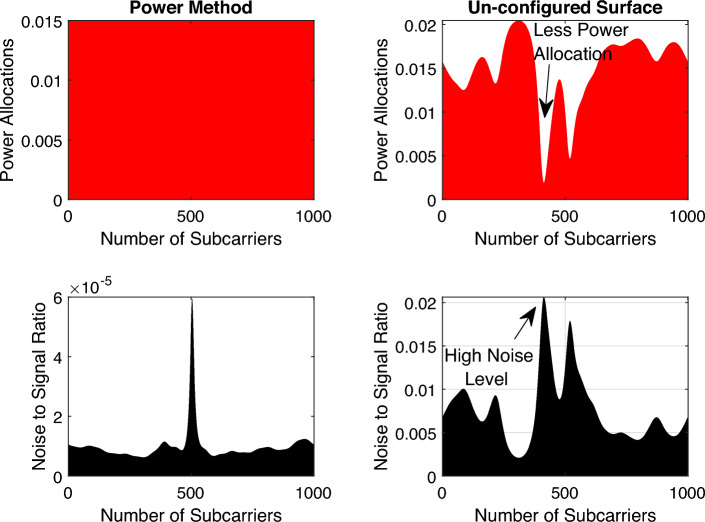
Power allocation for subcarriers in two cases: power method and un-configured surface. In the power method, the water-filling algorithm assigns the power equally for every subcarrier (nearly 0.015 Watt per subcarrier) due to the enhanced low noise level (in terms of $10^{-5}$), however, the noise is high in the un-configured case (0.02) where the algorithm allocates less power to the high noise level channel gain subcarriers and vice versa. Please refer to the black arrows in the figure.

The power distribution for all subcarriers $P_{0}, \ldots , P_{K-1}$ are satisfying $P=\frac{1}{K} \sum _{v=0}^{K-1} P_{v}$ where, $P_{v}=E\left\{ |\bar{s}[v]|^{2}\right\}$ is the power provided to subcarrier *v*. The notation $E\{.\}$ is the expectation operator. The power allocation for the subcarriers can be optimized by the water-filling algorithm^8^.

21 $$\begin{aligned} P_{v}=\max \left( \eta -\frac{B N_{o}}{\left| f_{v}^{H} h_{d}+f_{v}^{H} U^{T} w_{\theta }\right| ^{2}}, 0\right) , \end{aligned}$$

where, the parameter $\eta \ge 0$ is chosen to satisfy $\frac{1}{K} \sum _{v=0}^{K-1} P_{v}=P$. The water-filling algorithm is used to allocate the transmitted power fairly to all subcarriers. In Fig. 3, we show the comparison of the power allocation between the power method and the RIS surface when it is un-configured (replaced by a metallic sheet) to demonstrate how the algorithm allocates the power to each subcarrier taking into consideration the channel status. The noise level is very low in case of the power method (in terms of $10^{-5}$) while it is high in the un-configured surface case, consequently, the water-filling algorithm will assign the power to each subcarrier taking into consideration the noise level e.g., refering to the arrows in Fig. 3, the water-filling algorithm assign less power to the subcarriers with bad channel status and vice versa.

### Computational complexity

This section addresses the level of computational complexity involved in the proposed optimization method of the RIS phases and subcarriers power allocation. We approximated the $\mathcal {O}$ complexity of the suggested algorithms by using the same method in^12^. In the power method, the term $h_{\mathrm {equivalent}}$ needs $(N+1)^{2}$ complex multiplications, whereas, the term $h_{\mathrm{ equivalent }}\Omega _{i}$ needs $(N+1)$ complex multiplications and phases quantization need *N* complex computations. Moreover, assigning power to all subcarriers requires $2(K\times M)$ complex multiplications. Therefore, overall approximated complexity per iteration is given as $\mathcal {O}_{\mathrm {Power}}$:

22 $$\begin{aligned} \mathcal {O}_{\mathrm {Power }}=\mathcal {O}\left( \left( (N+1)^2+(N+1)+N\right) I_R+2(K M)\right) , \end{aligned}$$

where, $I_{R}$ is denoted as the maximum number of iterations that are needed to achieve the optimal data rate. In the SDR method, the complexity can be determined by calculating the complex operations of the following terms. The terms $\mathfrak {Q}$ and $\phi$ in Eq. (19) need $(N+1)^2$ complex multiplications. $(N+1)^{4.5}$ complex multiplications are required by the Gaussian randomization method to compute the best solution $\phi ^*$. In addition, $2(K\times M)$ complex multiplications are required for subcarriers power allocation computation. As a result, the overall approximated complexity is given as $\mathcal {O}_{S D R}$:

23 $$\begin{aligned} \mathcal {O}_{SDR}=\left( 2(N+1)^2+(N+1)^{4.5}+2(K\times M)\right) . \end{aligned}$$

The $\mathcal {O}_{SDR}$ in (23) is very similar to the results provided in^8^ and^6^ where the complexities are $\mathcal {O}\left( N K I_{S A}+N^{4.5} K^{3.5} I_{T}\right)$ and $\mathcal {O}(N+1)^{4.5}$, respectively. $I_{S A}$ denotes the number of iterations that the successive approximation requires and $I_{T}$ is the total time that is needed to solve the problem during the successive convex approximation algorithm. The run elapsed time in Table 1 is calculated using the tic toc function available in MATLAB. Complexity and run time are compared between the power method and the SDR for different number of elements *N* as presented in Table 1. For instance, if we consider the lowest number of elements (100) in Table 1 and compute the complexities taking into consideration the number of iterations, elements, subcarriers and channel taps. We notice that the complexity in the power method is in terms of thousands of operations while in the SDR is in terms of millions. Furthermore, the SDR method takes more run-time than the power method under different RIS sizes.

**Table 1:** Complexity and run-time comparison for different values of *N*.

Number of elements *N*	$\mathcal {O}_{\mathrm{ Power } }$	$\mathcal {O}_{S D R}$	Power run-time	SDR run-time
100	$0.6 \times 10^6$	$1.046 \times 10^9$	46.2 s	83.914 s
225	$2.62 \times 10^6$	$39.2 \times 10^9$	91.6 s	287.045 s
400	$8 \times 10^6$	$518 \times 10^9$	105 s	3205 s

## Simulation results

In this section, we evaluate the performance of the achievable data rate using the power method and compare it with the state-of-the-art SDR method. The simulation parameters provide in Table 2 are considered based on the 3GPP channel model^13^. We consider an OFDM system with $K=1000$ subcarriers and a more realistic multi-path channel model with $M=23$ channel taps at random delays with an exponential power decay profile. The deployment locations for the AP, RIS and user are shown in Fig. 1. We consider LoS channels between the AP-RIS-User indirect link as per Fig. 1, to guarantee effective transmissions and large channel gains. However, the non-line-of-sight (NLoS) propagation is assumed between the AP and the user.

**Table 2:** Simulation parameters.

Parameter	Value
Carrier frequency $f_{c}$	3e9 Hz
Speed of light *C*	3e8 m/s
Wavelength $\lambda$	0.1 m
*N*	4096
Number of horizontal elements $N_{h}=64$	64
Number of vertical elements $N_{v}=64$	64
Vertical and horizontal element spacing	$\lambda / 4$
AP Location in meter, coordinates [*x*, *y*, *z*]	$$\left[ \begin{array}{lll}40-150&0\end{array}\right] ^T$$
User Location in meter, coordinates [*x*, *y*, *z*]	$$\left[ \begin{array}{lll}20&0&0\end{array}\right] ^T$$
RIS Location in meter, coordinates [*x*, *y*, *z*]	$$\left[ \begin{array}{lll}0&0&0\end{array}\right] ^T$$
Pathloss NLoS	$34.53+38 \log _{10}(d)$
Pathloss LoS	$30.18+26 \log _{10}(d)$
Channel taps *M*	23
Total number of subcarriers *K*	1000
Transmitted power *P*	15 W
Bandwidth *B*	15e6 Hz

For Comparison with power method, we consider the following benchmark schemes:

1. The SDR method.
2. The strongest and the weakest SNR configurations which are generated from the codebook $W_{\theta _{4 N}} \in \mathbb {C}^{N \times 4 N}$.
3. The random phase case where we assume that each RIS reflecting coefficient has a random phase independently and uniformly distributed in $[0\quad 2 \pi ]$ and the maximum amplitude, based on which we obtain the water-filling transmit power allocation.
4. Un-configured surface where we replaced the RIS with a passive metal sheet causing zero phase-shifts.

**Figure 4:**
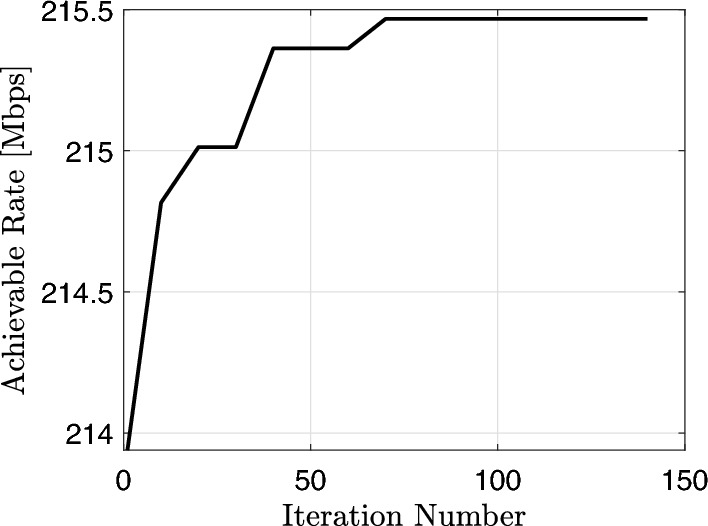
Convergence of Algorithm 1.

First, we evaluate the convergence behaviour of Algorithm 1. The number of RIS reflecting elements is set to $N=2500$ at SNR = 40 dB and 30 independent channel realizations. We notice that the achievable rate is generated progressively with higher values consistently. Figure 4 shows the achievable rate over several iterations. It is noticed that the minimum achievable value of the data rate is 214 [Mbps] while the highest one is 215.5 [Mbps] so, the difference is very small. Consequently, such a small difference in data rate values not only reflects the fast convergence but also validates the efficiency of the power method. We have initialized the power method from the strongest SNR configuration that has been generated for the $\left\{ W_{\theta _{4 N}}\right\}$ codebook which will, in turn, lead to higher data rate and small divergence values.

**Figure 5:**
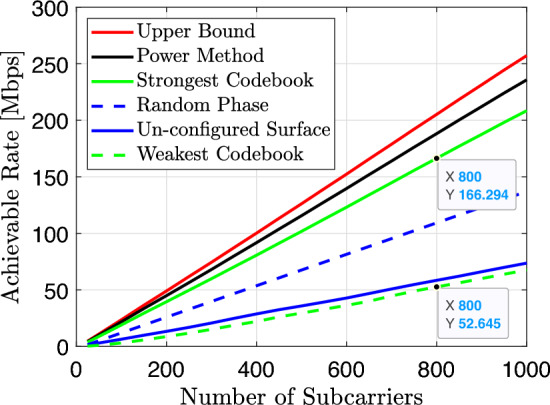
Comparison of the achievable data rate versus number of subcarriers for different schemes.

In Fig. 5, we compare the achievable data rate of the proposed power method with the upper bound, the strongest, weakest SNR configuration selected from the $\left\{ W_{\theta _{4 N}}\right\}$ codebook, random phase and lastly with the case when the surface is un-configured (a metal sheet case) for $N=4096$. The proposed power method outperforms both the strongest and weakest beamforming codebook, the random phase, and, the un-configured RIS case. The achievable data rate is dramatically boosted as the range of subcarriers increases. There is a gap between the rate achieved by the power method and that achieved by the beamforming codebook benchmark, and this gap gradually increases with the increase of number of subcarriers due to the increased channel frequency selectivity. However, the extended codebook $W_{\theta _{4 N}}=\left[ W_\theta ,-W_\theta , W_{(\theta , f l i p)},-W_{\theta , \mathrm {flip}}\right] \in \mathbb {C}^{N \times 4 N}$ provides varieties of configurations that range from the weakest to the strongest SNR configurations which are, in return, lead to data rate enhancement. For example, the achievable data rate from the weakest to the strongest codebook configuration at $N=800$ is around 120 [Mbps]. In comparison to un-configured RIS (metal sheet) configuration, the proposed power method approach improves the sum data rate by 2.5 order of magnitude.

**Figure 6:**
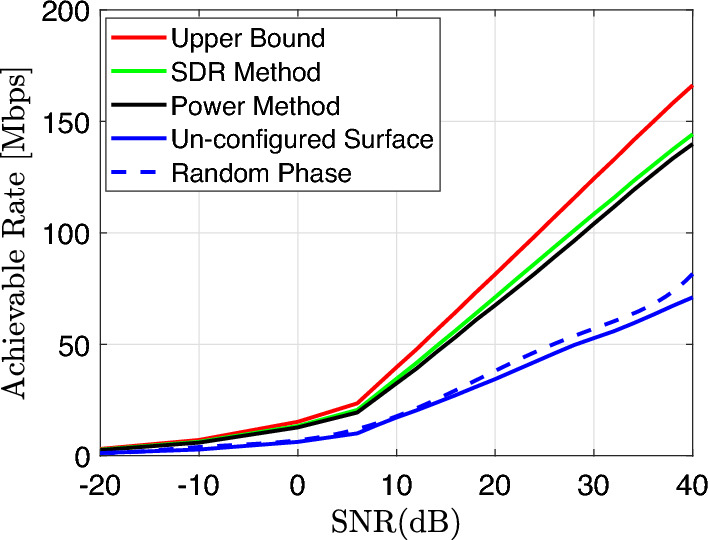
Achievable data rate versus SNR.

In order to validate the performance of the proposed method, Fig. 6 shows the performance of the iterative power method and the benchmark schemes at different SNR values, with $N=400$. It is noticed that all the schemes with RIS (SDR method, power method and random phase) outperform the scheme without RIS (un-configured surface), owing to the RIS-enhanced average channel power between the AP and the user. Additionally, the proposed iterative power method and the SDR scheme both significantly outperform the random phase scheme in terms of achievable rate because of the tuned RIS coefficients that help the direct and the reflecting channel to be superimposed more constructively at the receiver. The SDR method shows slightly superior performance to the power method at low and high SNR regimes. Nevertheless, the performance gap between them is very small and can be sacrificed for the sake of practical implementations and lower complexity. Table 1 reveals that complexity increases when the number of elements increases in both SDR $\mathcal {O}_{SDR}$ and power $\mathcal {O}_{\mathrm{ Power }}$ methods. However, it is shown that complexity and run-time consumption in the SDR method is higher than the power method. Our proposed method is simple, practical and less complex than other algorithms^6,8^, as it uses fewer number of operations.

**Figure 7:**
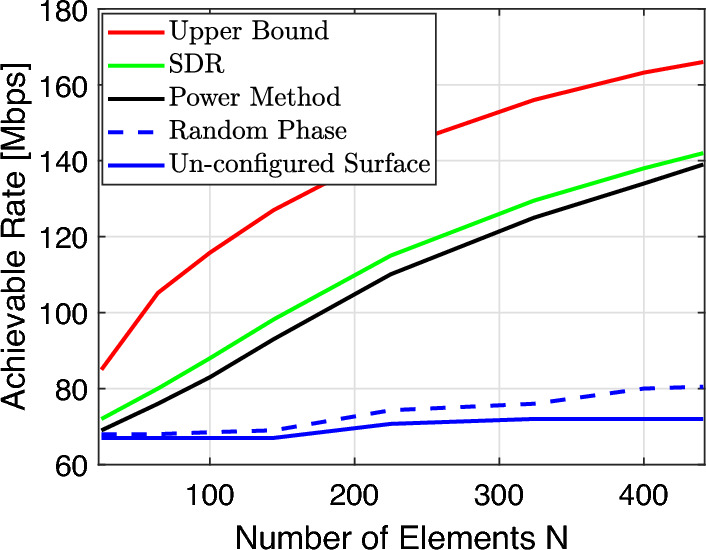
Achievable rate versus a number of reflecting elements *N*.

In Fig. 7, we compare the achievable rate of different schemes versus the number of RIS elements at SNR = 25 dB. Firstly, it is clear that the rate performance of the random phase shift scheme at the RIS is independent of the number of elements. This is to be expected since this method only has an aperture gain with no passive beamforming gain. Secondly, we can observe that both the SDR and the power method show superior performance as compared to the scheme with random phase shifts at the RIS and the un-configured surface. The achievable rate of the power method and the SDR keep increasing as the number of elements increases. This makes sense since the passive beamforming performance is gradually enhanced.

## Conclusion

In this paper, we showed that the RIS can be configured to provide significantly higher data rate, in comparison with un-configured surface, e.g., metal sheets. The codebook approach was implemented to generate varieties of phase configurations for the RIS surface in which we searched for the best configuration to be used in the optimization process. A problem formulation is described, and a solution is presented using the power method and the water-filling algorithm. To validate our proposed method, it is compared with the state-of-the art SDR scheme and the achievable data rates which are resulted from all methods are compared with the upper bound of the system. We noticed that the power method gives performance very close to the SDR method, however, our developed method shows less computational complexity and run-time.

## Data Availability

The data generated or analysed during this study are available from the corresponding author on reasonable request.
